# Brain malformations in diprosopia observed in clinical cases, museum specimens and artistic representations

**DOI:** 10.1186/s13023-023-02617-5

**Published:** 2023-03-16

**Authors:** Helga Rehder, Susanne G. Kircher, Katharina Schoner, Mateja Smogavec, Jana Behunova, Ulrike Ihm, Margit Plassmann, Manuel Hofer, Helmut Ringl, Franco Laccone

**Affiliations:** 1 Institute of Medical Genetics, Medical University of Vienna, Waehringer Strasse 10, 1090 Vienna, Austria; 2 Institute of Pathology, Fetal Pathology, Philipps-University of Marburg, Marburg, Germany; 3 Praxis fuer Praenatalmedizin, Dortmund, Germany; 4 Department of Biomedical Imaging and Image-Guided Therapy, Medical University of Vienna, Vienna, Austria; 5 Institue of Medical Genetics, Section Clinical Genetics, Medical University of Vienna, Waehringer Strasse 10, 1090 Vienna, Austria

**Keywords:** Diprosopus, Janiceps, Parasitic twin, CNS malformations, Holoprosencephaly, Cerebral di-symmetry, ICSI, Tlatilco culture, Schedels world chronicle, Paul Klee

## Abstract

**Background:**

Diprosopus is a rare malformation of still unclear aetiology. It describes a laterally double faced monocephalic and single-trunk individual and has to be distinguished from the variant Janus type diprosopus.

**Results:**

We examined seven double-faced foetuses, five showing true diprosopus, and one each presenting as monocephalic Janiceps and parasitic conjoined twins. Four of the foetuses presented with (cranio)rachischisis, and two had secondary hydrocephaly. Three foetuses showed cerebral duplication with concordant holoprosencephaly, Dandy-Walker cyst and/or intracranial anterior encephalocele. In the Janiceps twins, cerebral duplication was accompanied by cerebral di-symmetry. In the parasitic twins the cyclopic facial aspects were suggestive of concordant holoprosencephaly. In one of the true diprosopus cases, pregnancy was achieved after intracytoplasmic sperm injection. Whole-exome sequencing, perfomed in one case, did not reveal any possible causative variants.The comparison of our double-faced foetuses to corresponding artistic representations from the Tlatilco culture allowed retrospective assignment of hairstyles to brain malformations.

**Conclusion:**

Brain malformations in patients with diprosopus may not be regarded as an independent event but rather as a sequel closely related to the duplication of the notochord and neural plate and as a consequence of the cerebral and associated craniospinal structural instabilities.

## Background

The term ‘diprosopus’ describes a symmetrically and laterally double faced (πρόσωπο = face), monocephalic and single-trunk individual. It is defined by the duplication of at least two facial structures [1]. Diprosopus is considered a subtype of conjoined twins; in this context, it is also referred to as ‘parapagus diprosopus’. The Janus type of opposing facial duplication, occurring in ventrally fused ‘cephalothoracopagous’ twins, may be regarded as a monocephalic diprosopus in a broader sense of the term.

**Fig. 1:**
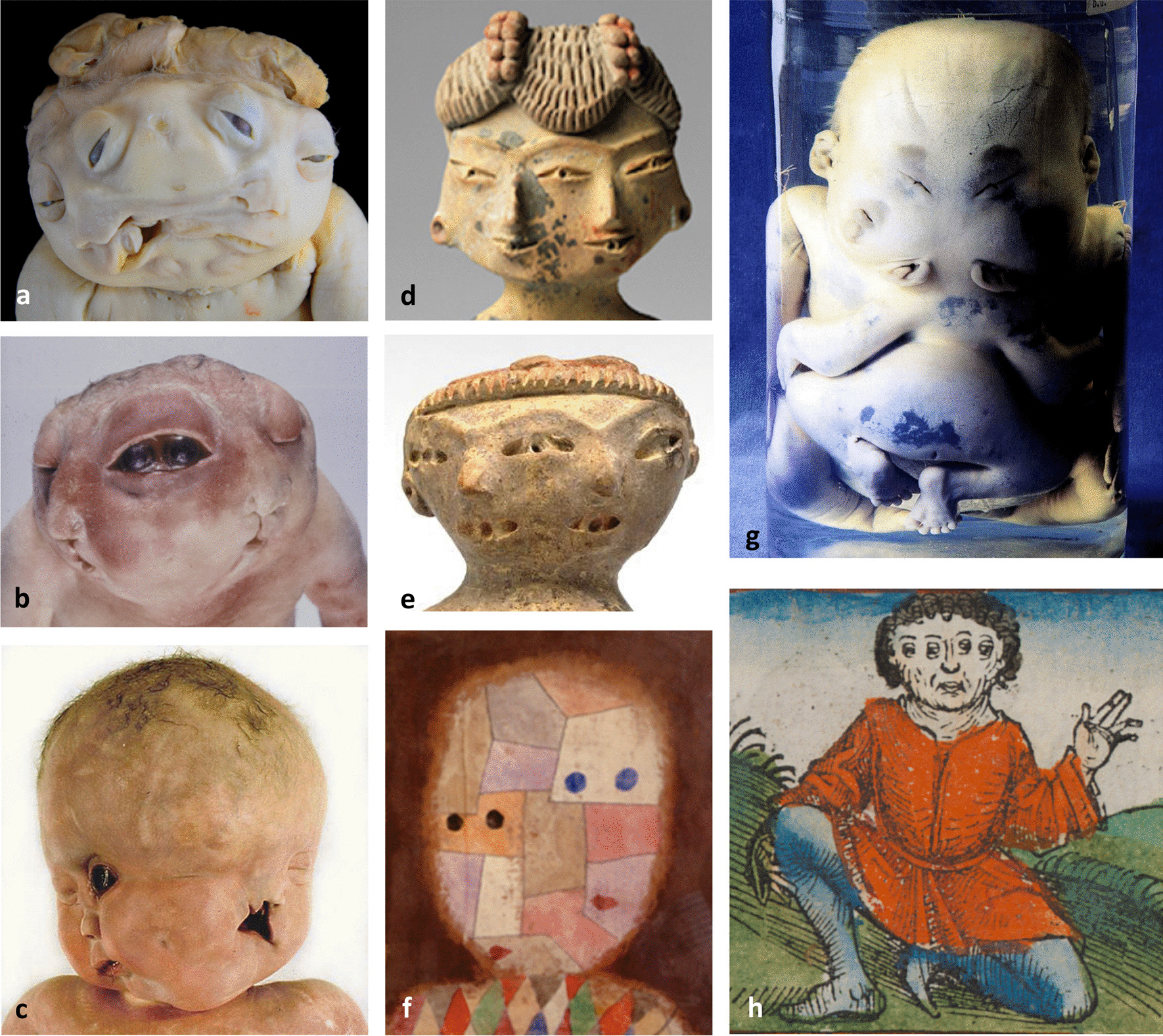
DIPROSOPUS: Exencephalic female exhibit (case 4) with diprosopus tetrophthalmus and discordant unilateral CLP (**a**). Anencephalic female diprosopus tetrophthalmus (case 1) with concordant contralateral CLP (**b**). Female diprosopus triophthahlmus (case2) with cebocephaly of the right face. The left face shows right-sided anophthalmia in the presence of a right palpebral fissure, right-sided arrhinia and double unilateral CLP (**c**). Double-faced female figurine 1200–900 B.C.E., early formative Tlatilco ceramic with traces of pigment, h. 9.5 cm, w. 4.8 cm, d. 2.1 cm (3 3/4 × 1 7/8 × 1 3/16 in.), Princeton University Art Museum; gift of Gillett G. Griffing 1999–245 (**d**). Double-faced female figurine 500–400 B.C.E., middle formative Tlatilco ceramic with pigment, h. 5.7 cm (2 ½ in), the Art Institute of Chicago. Gift of Ethel and Julian Goldsmith 2008–676 (**e**); Paul Klee’s diprosopus tetrophthalmus symbolizing the ‘Parent’s Image', watercolour on handkerchief on cardboard 1933 357, 45 × 37 cm, private property, USA (**f**). Case 7—Heteropagous parasitic single chest twins showing diprosopus with 4 ears, two mouths, arrhinia and two palpebral fissures, each with a lower lid notch), indicating cyclopic fusion (**g**). Schedel’s coloured print of a monocephalic man showing partial diprosopus with 4 eyes, 1 nose and 1 mouth (Schedel’s world chronicle 1493, Bavarian State Library Munich, Rar. 287, fol. 12v) (**h**)

The oldest preserved representations of a diprosopus originate from the pre-Columbian village Tlatilco in central Mexico at 1200–400 B.C.E. [2–4] (Fig. 1d + e) and from Schedel ‘s world chronicle 1493 C.E. [5] (Fig. 1h). Also in modern arts, we found a depiction of an apparent diprosopus by Paul Klee from 1933 (Fig. 1f). A total of 81 scientific reports on human facial duplications have been documented since 1642, 38 early ones cited by Barr [6], 33 more recent ones cited by Bidondo et al., [1] and 10 additional cases [7–16]. The prevalence rate of Diprosopus has been calculated to be 2 per 1,000,000 births in Argentinia, accounting for 10% of all conjoined twins [1]. The prevalence of conjoined twins worldwide is 14.7 per 1,000,000, with a rate of < 3% of diprosopus, of < 1% of Janiceps twins and of 3.9% of parasitic twins [17]. There is a female preponderance with a ratio of m:f = 1:1,4. In approximately half of the cases, diprosopus is associated with craniorachischisis. Other frequent anomalies are cleft lip and palate (CLP), bifid tongue, cerebral duplication, cardiac septal defect, diaphragmatic hernia and laterality defects [1]. Prenatal diagnosis of diprosopia by 2D- and 3D colour Doppler ultrasonography is possible at 12 weeks gestation [15]. Diprosopia is incompatible with prolonged extrauterine life.

The aetiology of diprosopus is unknown. Increasing numbers of conjoined twins (not including diprosopia) born after the Chernobyl accident (5 in 96,438 births), the lack of familial aggregation, and the 21 singletons fathered by the Siamese twins Chang and Eng Bunker support causative nongenetic factors [1, 18, 19]. The presumably higher incidence of diprosopia in Latin America and the induction of a facial double malformation by a higher expression of Sonic Hedgehog (Shh) in animal models would be compatible with the involvement of causative genetic factors [20]. Proposed pathogenic mechanisms are as follows: Lateral *fusion* of two monozygotic embryonic discs [21]; *fission* of a single embryonic disc with the formation of two notochords, and thus of two cephalic neural plates and of an extra medially located cranial neural crest [22]; *duplication* of the early primitive node as the signal emission centre for the neural plate, thereby causing cranial bifurcation of the notochord, the formation of two vertebral axes and of two neural plates [1].

We added five more patients with true diprosopus (clinical cases 1–3 and exhibits cases 4 and 5) and two double-faced monocephalic homopagous and heteropagous conjoined twins (clinical case 6 + exhibit case 7) to these observations under consideration of the accompanying central nervous system (CNS)-abnormalities and with reference to corresponding representations in art.. In case 3 singleton whole-exome sequencing (WES) was performed.

## Results

Clinical and morphological data are summarized in Table 1.

**Table 1:** Clinical and morphological data of presented cases

Clinical Data	**Case 1 (1977)**	**Case 2 (1986)**	**Case 3 (2013)**	*Case 4 ( 1920)*	*Case 5 (n.d.)*	**Case 6 (1980)**	*Case 7 (1790)*
Maternal age	**21**	**32**	**33**	*n.d.*	*n.d.*	**27**	*n.d.*
Gravida/Para	**2 / 2** **(1 healthy son)**	**2 / 2**	**2 / 1 healthy daughter** **(2**^**nd**^** pregnancy by ICSI)**	*n.d.*	*n.d.*	**2 / 1 healthy son**	*n.d.*
Parental ethnicity	**German**	**German**	**Turkish** **no consanguinity**	*Austrian*	*Austrian*	**German**	*Austrian*
Family history maternal/paternal	**polydactyly, dizygous twins / dizygous twins**	**inconspicious**	**inconspicious**	*n.d.*	*n.d.*	**inconspicious**	*n.d.*
Prenatal diagnosis	** X-ray: craniorachischisis**	** US: polyhydramnios, hydrocephaly**	** US: hydrocephaly, diprosopus, DH**	*n.d.*	*n.d.*	** US: cephalothoraco-pagous Janiceps twins**	*n.d.*
Gestational week	**35/stillborn**	**39**	**18 + 4**	~ *36*	*Near term*	**34**	*Near term*
Weight / length	**1200 g/32 cm**	**3350 g/55 cm**	**267 g/24 cm**	*? / 35 cm*	*? / 42 cm*	**1072 g / 35 cm**	*n.d.*
Karyotype / Sex	**female**	**female**	**46,XX / female**	*female*	*Male*	**female**	*Male*
Cranio-/Rachischisis	** + / C1-L2**	**−/−**	**−/−**	+ */* +	*−/* +	**−/−**	*−/* +
Diprosopus	** + **	** + ** **Discordant cebocephaly**	** + **	+	+	** + Janus type**	+ */ cc cyclopia, parasitic twins*
No. of eyes	**3 orbits, 4 eyebulbs**	**4 orbits, 3 eyebulbs,** **hypotelorism of right face** ** + right-sided crypt-ophthalmus of left face**	**3 orbits, 4 eyebulbs,** **microphthalmia of medial eyebulbs**	*4 orbits,* *4 eyebulbs*	*3 orbits, 4 eyebulbs,*	**4 orbits** **4 eyebulbs**	*2 orbits* *4 eyebulbs*
No. of noses	**2**	**2** **single nostril right nose +** **absent right nasal wing left nose**	**2**	*2*	*2*	**2**	*2*
No. of mouths / oral cavities / tongues	**2 / 2 / 2 dorsally fused,** **Single LA/TR/ESO**	**2 / 2 / 2 dorsally fused,** **Single LA/TR/ESO**	**2 / 2 / 2 dorsally fused,** **Single LA/TR/ESO**	*2 / n.d. / n.d.*	*2 / n.d. / n.d.*	**2 / 2 / 2 dorsally fused** **2LA/2TR/single ESO**	*2 / n.d. / n.d.*
No. of ears	**2 + single interfacial** **ear dimple**	**2 + single** **Interfacial ear dimple**	**2 + single** **Interfacial ear dimple**	*2* + *single* *Interfacial ear dimple*	*2* + *single* *Interfacial ear dimple*	**4**	*4*
Cleft (lip) palate (C(L)P)	**cc contralateral CLP**	**CP right face + unilateral** **CLP left face**	**-**	*dc unilateral CLP right face*	*cc contralateral CLP*	** − **	* − *
Cerebral duplication	**?**	** + **	** + **	*?*	*n.d.*	** + **	*n.d.*
Cerebellar duplication	**?**	** − **	** − **	*?*	*n.d.*	** + **	*n.d.*
Cerebral malformation	**Anencephaly,** **Craniorachischisis**	**Hydrocephaly + HPE, lobar right, alobar left brain + sphenoidal MEC**	**Dandy-Walker cyst,** **Vermis hypoplasia,** **Clival MEC**	*Exencephaly,* *Craniorachischisis*	*Hydrocephaly*	**cc opposite right-angled outward rotation of frontal lobes**	*n.d* *HPE suspected*
Facial bones	**(Part.) Dup frontal, orbi-tal, temporal, mandi-bular + maxillary bones**	**(Part.) Dup. frontal, orbital, temporal, mandibular + maxillary bones**	**(Part.) Dup. frontal, nasal, orbital, temporal, mandibular + maxillary bones**	*(Part.) Dup nasal, orbital, mandibular* + *maxillary bones, fused medial ZP*	*(Part.) Dup. of facial bones, no fusion of ZP*	**Duplicated**	*n.d.*
Skull base	**(Part.) Dup. of fossa ant. + ethmoidal + sphe-noidal bones, sep. PF**	**(Part.) Dupl. of fossa ant. + sphenoidal bones + fusion of PF**	**(Part.) Dup. of fossa ant., ethmoidal + sphenoidal bones, separate PF**	*Medially incompl. dup. of skull base, separate PF,* *2 foramina magna*	*(Part.) Dup. of fossa ant., ethmoidal* + *sphenoidal bones,*	**cc opposite right-angled outward rotation of anterior fossae halves**	*n.d.*
Duplic. of vertebral bodies	** + **	**C1—L2**	**C1-TH6**	*C1-L5*	*C1—L5*	**n.d**	*n.d.*
Associated malformations	**TOF + DH + SUA**	**dc cebocephaly + low set VSD**	**ASD2 + DH**	*n.d.*	*n.d.*	**SGIT/OC/cc CHD/dc DH**	*dc AA/SB/OC/single UC in the autosite*

*Bold* clinical cases

italic exhibits

*AA* anal atresia; *ASD* atrial septal defect; *cc* concordant; *CHD* congenital heart defect; *CVA* caudal vermis aplasia; *dc* discordant; *DH* left-sided diaphragmatic hernia; *DWC* Dandy-Walker cyst; *ESO* esophagus; *HPE* holoprosencephaly; *LA* larynx; *MEC* meningoencephalocele; *OC* omphalocele; *PF* pituitary fossa; *SB* spina bifida; *SGIT* partially single gastrointestinal tract; *SHC* single hypoplastic cerebellum; *SUA* single umbilical artery; *TOF* tetralogy of Fallot; *TR* trachea, *UC* umbilical cord; *US* prenatal ultrasonography; *VSD* ventricular septal defect of the heart; *ZP* zygomatic processes

### General features in cases 1–5

**Fig. 2:**
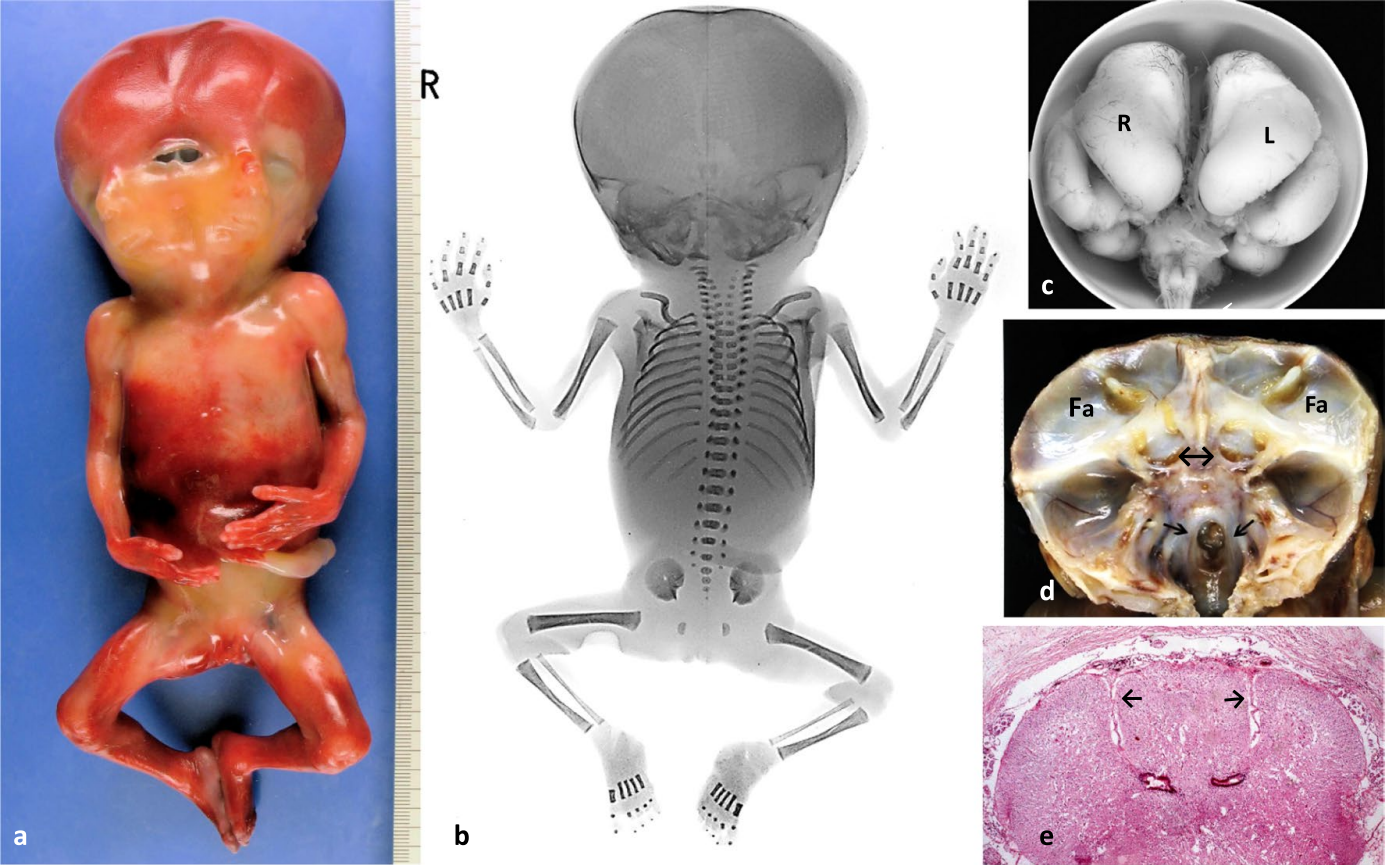
Pathological findings in diprosopus case 3. Female fetus at 18 weeks showing diprosopus tetrophthalmus with median microphthalmic eye bulbs and a single median ear pit (**a**); X-ray showing mandibular duplication, splicing of the cervical spine and duplication of thoracic vertebral bodies T1-T7 (**b**); basal view of the duplicated right (R) and left (L) forebrain with two hemispheres each and a single cerebellum ( ←) (**c**); skull base presenting duplication of frontal fossa (Fa) with two ethmoidal crests, two pituitary fossae ( ↔), each with 2 optic nerves, and a clival cleft (↘↙) (**d**); histologic section of the thoracic spinal cord showing incomplete dimyelia with two central canals and two ventral fissurae medianae (→ ←) (e)

*Cases 1–5* displayed true monocephalic single trunk diprosopus with duplication of the forehead, eyes, nose, mouth and chin, but not of the ears with the exception of an additional common ear pit at the interfacial midline (Fig. 1a-c, 2a, 4d). There were four orbits with four eye bulbs in the foetus in *case 4*. The foetuses in *cases 1, 3 and 5* presented fusion of the medial orbits but separated eye bulbs, associated with microphthalmia in case 3. Concordant contralateral CLP was found in foetuses in *cases 1 and 5*, while that in *case 4* showed discordant unilateral CLP. Unilateral facial clefts were medially located, and all foetuses showed microstomia and microgenia. *Case 2* requires special mention. Its right face displayed classical cebocephaly with hypotelorism, a proboscis-like nose with a single nostril, microstomia, and a cleft palate, whereas the left face appeared non-cebocephalic. It presented with pronounced hypertelorism, right-sided anophthalmia in the presence of a right palpebral fissure, a nose with only one left nasal wing and nostril, and doubled right-sided CLPs (Fig. 1c). Autopsies of the foetuses in *cases 1–3* showed dorsal fusion of the oral cavities, tongues, and pharynges, a single larynx, trachea and oesophagus as well as single internal organ systems. In addition, we found cardiac defects and left-sided diaphragmatic hernias (Table 1).

### CNS, skull and skull base in cases 1–5

In the foetuses in *cases 1 and 4* there was anencephaly and exencephaly, respectively, associated with craniorachischisis down to L5 (Fig. 1b + a; and 4a). The skull base in the foetus in case 1 showed duplication of the anterior cranial fossa, including the ethmoidal and partly the sphenoidal bones, and a cleft foramen magnum (case 4, see 3D-CT).

**Fig. 3:**
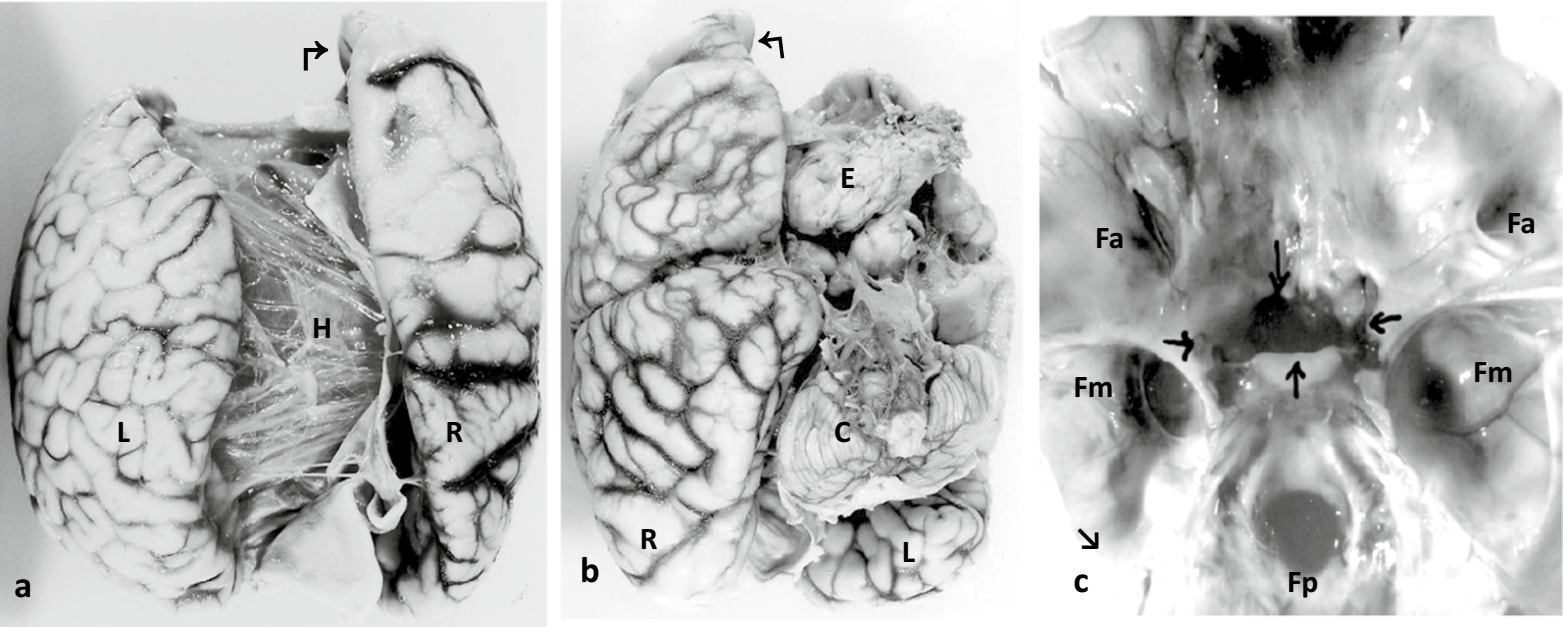
Neuropathological findings in diprosopus case 2—Top view of the brain, showing alobar holoprosencephaly (HPE) of the left forebrain (L) and lobar HPE with incomplete interhemispheric fissure (↱) of the right forebrain (R), and also internal hydrocephaly (H) (**a**); basal view of the cerebrum associated with the right cebocephalic face, presenting lobar HPE (R) with incomplete interhemispheric fissure (↰), anterior encephalocele (E) and a single cerebellum (C). (**b**); skull base presenting duplicated 2 anterior cranial fossae (Fa) with only a single left ethmoidal crest (↘) in the presence of two, left and right olfactorial canals. The 2 pituitary grooves are fused and cleft in the depth (↓↑), thus enabling prolaps of a frontal encephalocele. There is one optic fossa on each side of the fused pituitary grooves (→ ←), the left one showing cross section of one optic nerve and the right one harboring two optic nerves. There are normal two middle (Fm) and one posterior cranial fossae (Fp) (**c**)

In the foetus in *case 2,* assessment of the CNS was complicated by hydrocephaly and by a large intracranial sphenoidal meningoencephalocele (MEC) (Fig. 3a + b). There was cerebral duplication with a discernible interhemispheric fissure on the convexity of the right cerebrum showing incomplete division in its depth and thus corresponding to 'lobar holoprosencephaly' (HPE). Total lack of an interhemispheric fissure in the non**-**lobulated left brain indicated 'alobar HPE'. The ventricular systems were dilated and cystic, resulting in internal hydrocephaly. There was a single cerebellum and a partially duplicated brainstem and spinal cord. The skull presented duplication of the frontal bones and the anterior fossa with a lack of ethmoidal structures in the anterior fossa related to the cebocephalic face, while in the left anterior fossa a short ethmoidal crest was recognizable. A longitudinal bone plate, corresponding to angulated medially fused greater wings of the duplicated sphenoidal bone, separated the anterior fossae. The sphenoid bodies were broadened, and the pituitary grooves fused and split at the bottom ventral to a single fused dorsum sellae, thus representing the site of the sphenoidal MEC (Fig. 3c). There was 1 optical canal on each side of the cleft common pituitary groove; the right one showed cross sections of two optic nerves, and the left one contained one left optic nerve. There was duplication of the cervical and thoracic vertebral bodies.

The foetus in *case 3* presented duplication of the cerebrum (Fig. 2c); of the mesencephalon with two aquaeducts; and partial duplication of the pons, medulla and of the cervical and upper thoracic spinal cord, as indicated by two central canals, two ventral fissurae medianae (Fig. 2e) and two dorsal sulci mediani, and a single cerebellum. There were hypoplasia of the single vermis with the single fourth ventricle dorsally opening into a small Dandy-Walker cyst and a small clival MEC, covered by pharyngeal mucosa. Accordingly, the skull presented four frontal bones and the skull base had two anterior cranial fossae, the medial parts being slightly smaller. Two ethmoidal crests indicated duplication of the ethmoidal bone. The sphenoid bone was partially duplicated and medially fused with two capped inner wings forming the boundary between the two anterior fossae, a distinctly widened sphenoid body and two separate pituitary grooves, each harbouring a pituitary gland and two optic canals. A caudal cleft of the clivus represented the site of the small anterior MEC (Fig. 2d). The upper vertebral bodies of the cervical and upper thoracic spine were broadend with duplicated ossification centres on X-ray images (Fig. 2b).

**Fig. 4:**
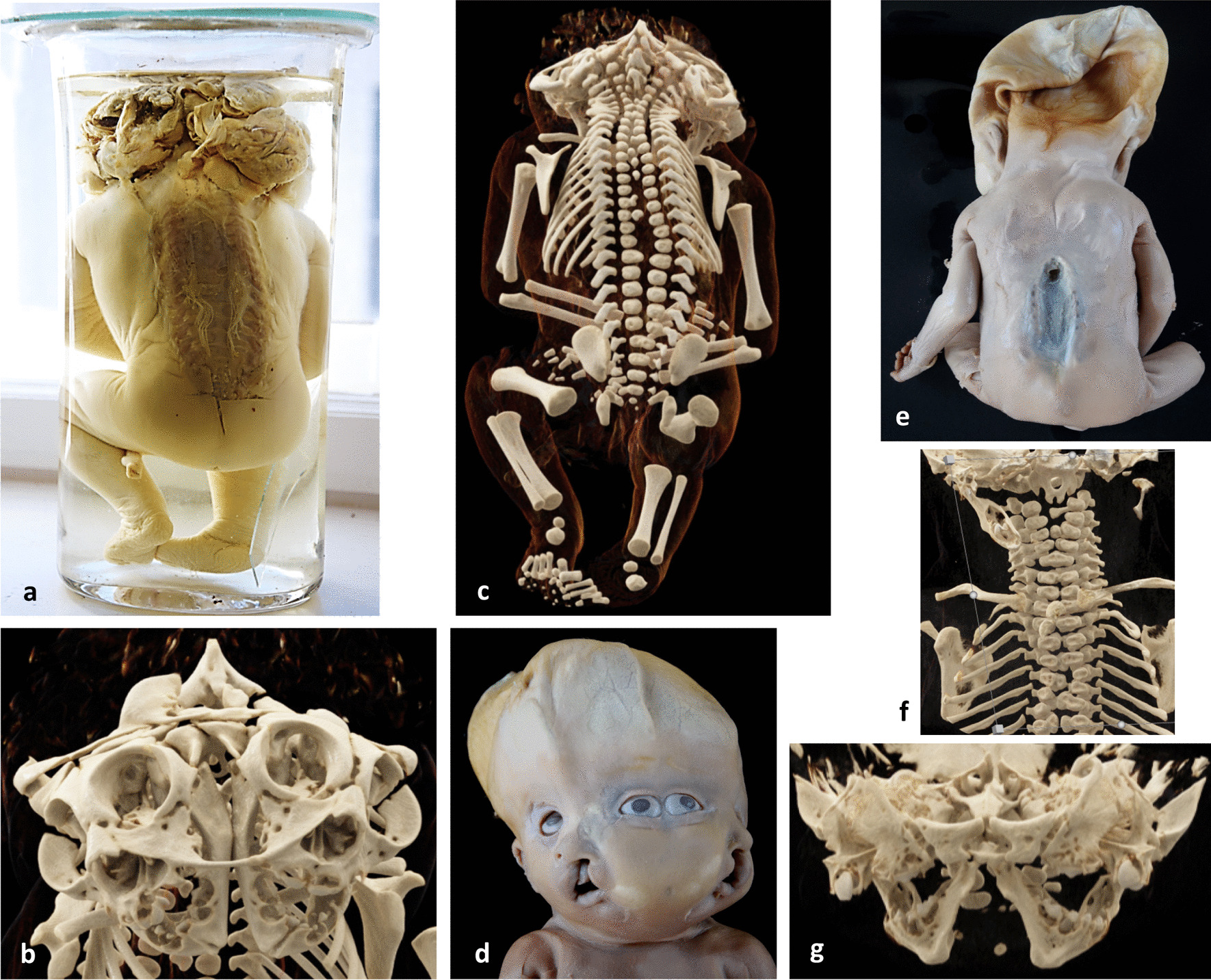
Case 4-Dorsal view of a female 3D-CT images of cases 4 and 5. Dorsal view of female diprosopus showing exencephaly and extensive craniorachischisis (**a**); frontal view of the faciial 3D-CT, showing acrania, osseous spike in between the duplicated skull base, duplication of facial bones, partial defects of median temporal bones and handle-shaped interfacial midline fusion of the median zygomatic processes (**b**); dorsal view of the fetal skeleton in 3D –CT displaying craniorachischisis, duplication of vertebral bodies down to L5 and splitting of the upper cervical spine (**c**). Case 5—Frontal view of the face of a male diprosopus terophthalmus with fused orbits, median ear pit and contralateral CLP (**d**); dorsal full length immage displaying collapse of the skull due to preexisting hydrocephaly and thoracolumbar rachischisis (**e**); frontal view of the upper spine in 3D-CT showing duplication of vertebral bodies, but not of vertebral arches and single foramen occipitale magnum (**f**); frontal view of facial 3D-CT presenting duplication of facial bones, defect of the inner temporal bones and narrowing, but no fusion of the zygomatic processes (**g**)

The foetus in *case 5* showed isolated rachischisis extending from Th8 to L5. The brain had been removed, but extensive dilatation and folding of the scalp indicated preceding hydrocephaly (Fig. 4e).

### 3D-CT in cases 4 and 5

In the foetus in *case 4*, 3D-CT confirmed craniorachischisis and revealed duplication of vertebral bodies down to L5 and complete splitting of a V-shaped cervical spine with duplication of the transverse vertebral processes from C1 to C4 (Fig. 4c). The separate spinal canals, thus formed, ended in two separate and cleft foramina magna. Duplication of the skull base involved the anterior cranial fossa, the ethmoidal bone, part of the sphenoid bone and the middle and posterior cranial fossae. This finding was indicated by a bony spike between the two cleft foramina magna and a separating incomplete bony ring surrounding each of the two skull bases (Fig. 4b). It is composed of basal sections of the apically defective frontal, parietal and occipital bones. Facial duplication affects the orbital, nasal, maxillary, mandibular and zygomatic bones with the two medial zygomatic processes handle-like connected at the interfacial midline (Fig. 4b).

In the foetus in *case 5*, we found duplication of the vertebral bodies with divergence at the site of the rachischisis and splitting of the upper cervical spine from C1 to C4 (Fig. 4f). The skull had been dissected. The skull base demonstrated duplication of the anterior fossa, ethmoidal and partly sphenoid bones, including the sella, and a single foramen magnum. In the frontal view, we observed duplication and medial fusion of the orbits, harbouring two recognizable optic nerves. There was duplication of nasal, zygomatic, maxillary and mandibular bones. The upper jaws showed concordant medial clefts. The medial temporal bones were not recognizable. The medial zygomatic processes were not fused at the interfacial midline. The medial mandibular rami and medial mandibular joints were hypoplastic (Fig. 4g).

### Features of additional cases 6 and 7

**Fig. 5:**
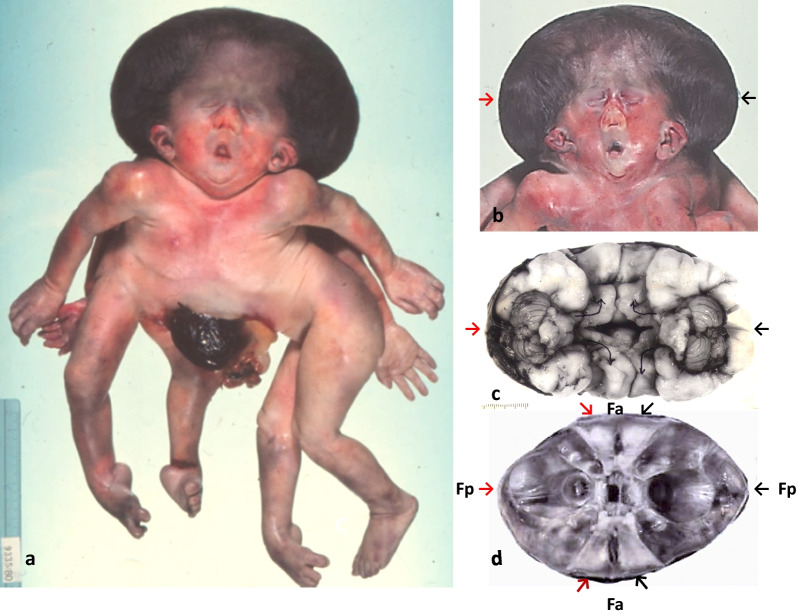
Craniocerebrothoracic di-symmetry in Janiceps twins case 6. Janiceps twins with two opposing faces and chest fronts and facial midline defects, (**a** + **b**); basal view of the two brains with two opposing cerebellums (→ ←) and the four hemispheric frontal lobes (↴) turning at right angles into the right and left sided half of the opposing fused anteriorfossae *(↘↙—↗↖) (****c*** + **d**); top view of the cranial bases showing two opposed posterior fossae (→ ←) and, at right angles to these, two opposed frontal fossae, (↘↙—↗↖) (**d**)

*Case 6* represents frontally fused symmetric cephalothoraco(homo)pagous female twins with Janus-type diprosopus, separated respiratory, cardiac, hepatic and urogenital tracts, and fused lower pharynges, oesophagus, stomach and small intestine, but with two papillae Vateri. There was a communication between a persistent left aortic arch of one twin and the descending aorta of the co-twin. Frontal cephalothoracic fusion resulted in two opposing skulls and vertebral axes and two opposing faces and chest fronts, located lateral to the skull (Fig. 5a + b). Frontal fusion of skull bases and ventricular systems and right-angled outward rotation of the halves of the anterior cranial fossae and the cerebral frontal lobes resulted in cranial and cerebral di-symmetry (Fig. 5c + d). Frontal fusion of the chests resulted in medial splitting of the ribcages, outward rotation of their halves and fusion with the co-twin’s chest halves and thus in thoracic di-symmetry. Hearts, respiratory systems, and upper abdominal organs were oriented according to the facial planes and aortae and urogenital organs according to the vertebral axes. The singular upper gastrointestinal system showed a central position. There were discordant omphalocele, a left-sided diaphragmatic hernia and a ventricular septal defect assigned to one frontal plane and tetralogy of Fallot to the other; and discordant scoliosis, exstrophy of the lower urinary bladder, hydronephrosis, assigned to one vertebral axis (Table 1). The two umbilical cords displayed common insertion into a common placenta.

*Case 7* entailed cephalothoraco(hetero)pagous male twins with the lower body half and the arms of a distinctly smaller twin (parasite) frontally attached to the epigastric region of a normal sized co-twin (autosite). The neck and thorax of the parasite were missing. The single head showed diprosopia with laterally fused equally sized faces and with duplication of the mouth, chin and ears (Fig. 1g). Noses were missing, and the two palpebral fissures showed a median lower lid notch and included two fused eyeballs each, thus corresponding to concordant cyclopia and presumably HPE. In the autosite discordant spina bifida, imperforate anus and a small omphalocele containing intestinal segments of both twins and showing insertion of a single umbilical cord were mentioned in a Latin descriptio**n**.

### WES results in case 3

Variants were filtered based on minor allele frequency (MAF) using our in-house database, including data from more than 1000 whole exomes and published disease-causing variants. The remaining variants were filtered for genes associated with HPO terms based on foetal malformations with predefined criteria, namely, read depth > 9, current variant allele frequency of 0.19 and Phred scale base quality score > 99, to allow the detection of possible mosaicism. In the last step, data analysis was extended to the whole exome, where non-OMIM disease-associated genes were analysed. Special focus was based on genes associated with the Sonic Hedgehog pathway. No possible causative variant could be detected in the analysed genes. An analysis of variants in possible candidate genes was because of unavailable parent samples not sufficiently possible.

## Discussion

A variety of major CNS malformations were observed in seven monocephalic double-faced foetuses, five presenting with true single-trunk diprosopus and two presenting as Janus-type and parasitic cephalothoracopagous twins, respectively (cases 6 and 7). CNS malformations comprised craniorachischisis with anencephaly and exencephaly (cases 1, 4), spina bifida (cases 5, 7), secondary hydrocephaly due to HPE and to spina bifida (cases 2, 5), cerebral duplication (cases 2, 3, 6), a Dandy-Walker cyst due to hypoplasia of the vermis (case 3), anterior encephalocele (cases 2, 3), HPE (cases 2, 7) and cerebral di-symmetry (case 6).

With respect to the prevalence rate of ***NTDs*** of 1:538 worldwide with 38.7% of NTDs being anencephalies [21, 23], the high rate of almost 50% of true diprosopus cases showing ex- or anencephaly among cases from our study and from the literature (22 anencephalies out of a total of 48 accessible diprosopus cases) makes a random association unlikely. However, the observed CNS malformations should be seen in the context of the accompanying craniospinal abnormalities. Duplication of the forebrain and part of the midbrain, pons, medulla, and spinal cord, thought to result from duplication of the early primitive node, notochord, and cephalic neural plate [1, 21, 22] are associated with duplications of bones particularly of the frontal skull base, the cervical spine, and of the thoracic and lumbar vertebral bodies. Corresponding bone anomalies were also seen in double-faced foetuses with craniorachischisis, suggesting a similar pathogenesis, regardless of whether the duplicated CNS tissues are preserved or perished in the absence of a sheltering skull and spinal canal. This makes cerebral duplication an obligatory feature of diprosopia. The high risk for cranial NTDs in diprosopia could be explained by the problems with neural tube closure caused by incomplete duplication of still-fused neural plates in the presence of a single extra medial neural crest [22] at an initiation site of the bidirectionally proceeding neural tube closure [24]. This is underlined by a low risk for anencephaly in dicephalic single trunk foetuses showing duplicated and separated spines and spinal cords.

Congenital *anterior meningoencephaloceles* (MECs) are rare in Western countries (1:35 000 live births). The majority of cases are not attributable to a classical NTD but rather represent a herniation through a defect or focal structural instability of the skull base [25]. The site of the larger intrapharyngeal MEC in case 2 represented fused and at the bottom cleaved pituitary fossae between duplicated sphenoid bones, and pituitary fossae fusion was facilitated by frontal angling of the medial sphenoid wings (Fig. 3c). The MEC contained cerebral tissue of the left holoprosencephalic brain and presumably irritated the development of the left face, causing displacement of its right eye socket and ipsilateral anophthalmia (see below; Fig. 1c). In the foetus in case 3, a smaller retropharyngeal MEC was associated with a midline defect in the lower ‘occipital’ clivus, and the upper ‘sphenoidal’ clivus was duplicated, thereby leading to osseous instability through widening of the sphenooccipital junction (Fig. 2d). In both of these foetuses the anterior intracranial MEC was thus closely related to the duplication of cranial bones, suggesting an association of diprosopus with a higher risk of anterior MECs. However, an anterior MEC has only been reported once in a patient with diprosopus. This patient had a large extracranial MEC extruding from a midline aperture between duplicated frontal bones and anterior fossae. It was associated with an extended interfacial cleft and–similar to our case 2—with discordant medial anophthalmia. Notably, a clival encephalocele has been observed in association with duplications of the pituitary, sella, ventricular system, basilary artery, tongue, frontal teeth and cervical vertebral bodies in one case, [26]. Other examples of small anterior MECs associated with diprosopia may have been missed in the past.

*HPE* is the most common forebrain malformation accounting for 1:7,500 livebirths and 1:250 early embryos [27, 28]. According to the degree of noncleavage into the two hemispheres, HPE is classified into an alobar, semilobar and lobar type, not considering HPE-microforms. HPE is associated with facial midline defects, such as cyclopia, ethmocephaly, cebocephaly or just hypotelorism, hypoplastic nose and CLP, and the severity of these features largely correlate with the HPE type. Following DeMyer’s statement ‘*the face predicts the brain’,* we may attribute concordant cyclopia in our case 7 to concordant alobar HPE, even though the brain was not exploitable [29, 30]. However, in case 2, this statement has only limited validity. Discordant cebocephaly of the right face was associated with lobar HPE, while left-sided alobar HPE did not result in cebocephaly or any other typical facial midline defect, possibly due to interference with an intracranial MEC. Three cases of severe facial midline defects in association with true diprosopus have been observed thus far—in addition to our case 2 resulting in 4 HPEs out of 48 accessible diprosopus cases. In one of two cases presenting with discordant cyclopia, autopsy had been declined [13]. In the second case in which the individual showed cyclopia on the left face and a normal mouth, nose and right eye, but anophthalmia with a depression at the site of a lacking left palpebral fissure on the right face, the brain had been described as having 'two hemispheres'—although two 'holospheres' would have been the more likely diagnosis as in cas 2 [31]. In a diprosopus with concordant cyclopia, HPE was confirmed but not specified [12]. However, in this case, the first HPE panel analyses of a diprosopus had been performed with sequencing of SHH, ZIC2, SIX3 and TGIF1 genes. Causative variants were excluded. This might suggest that HPE in diprosopia developed independently from an addtional pathogenic HPE gene variant.

The first *WES* in diprosopus, as performed in case 3, concerning a nonholoprosencephalic foetus of nonconsanguin**e**ous parents, did not reveal any causative variants, either in known disease-causing genes, or in known genes involved in the Shh pathway. A single ‘singleton WES ‘ does not, of course, allow the exclusion of a monogenic disorder, a genomic rearrangement or of somatic mosaicism. However, along the way, it may be possible that future technologies will reveal a genetic contribution to this developmental disorder. A careful molecular testing including ‘trio WES ‘ and ‘long-read whole-genome sequencing' is therefore warranted, even though the likelihood of finding a monogenic cause is very low. However, in *our* case *3*, pregnancy was achieved after intracytoplasmic sperm injection (*ICSI*). It has been shown that artificial reproductive techniques are associated not only with a twofold increase in congenital malformations [32] but also with a two- to threefold increase in monozygotic twin pregnancies after single embryo transfer. The use of frozen and thawed embryos—not applicable to our case 3—and zona pellucida manipulations, such as artificial handling or ICSI have been discussed as possible risk factors [33]. This may favour a mechanically conditioned non**-**genetic pathogenesis of diprosopus.

The cephalothoracopagous *Janus-type diprosopus* in case 6 differs from the parapagous diprosopus in presenting two bodies and ’frontal’ fusion. The two anterior fossae of the fused cranial vaults, the two faces and the two chest fronts were split into halves; each half turned outward at right angles and fused to the contralateral half of the co-twin's anterior fossa, face and chest. This created cranial and thoracic di-symmetry with crossing median axes. The two opposing faces thus located lateral to the vertebral axes showed symmetrical midline defects with marked hypotelorism, hypoplastic nose, microstomia and microgenia. These features are quite common in di-symmetric Janiceps twins [34, 35]. More common, however, is facial asymmetry, ranging from discordant cebocephaly, cyclopia or synotia up to mono-symmetrical single-faced cephalothoracopagous twins [34–39]. Consistent with the di-symmetry of the cranial vaults in the foetus in case 6, there was cerebral di-symmetry with the two frontal lobes exhibiting right-angled outward rotation and pairing with the co-twin’s frontal lobes (Fig. 5c). Cerebral di-symmetry has not been described previously and can as best be guessed from published ultrasound and MRI images [35–37]. Facial midline defects are generally related to HPE due to failure of forebrain cleavage into two hemispheres. However, this explanation does not hold for the Janiceps twins, since each set of frontal lobes originates from two different forebrains. This may indicate a different and non**-**genetic pathogenesis of midline defects in Janiceps twins being due to forebrain fusion instead of failure of forebrain fission. Frontal fusion of the brains in Janiceps twins is frequently mentioned in the literature, but we did not find illustrations showing secondary fusion of the two heterogenic frontal lobes in cases of Janiceps midline defects with the exception of a graphic representation [35].

*Parasitic heteropagous twins* represent asymmetric conjoined twins with a defective smaller and nonviable 'parasite' attached to and supplied by a near normal viable 'autosite'. In many cases, the site of attachment is the epigastric region, as in case 7, concerning the parasite’s hypoplastic pelvis and lower limbs, eventually including defective upper limbs and with rudimentary internal organs enclosed in the host’s thoracic cavity [41, 42]. Most parasitic twins are a-cephalic and a-cardiac. This finding is reminiscent of the a-cephalic and a-cardiac umbilicopagous cotwin in the twin-to-twin reversed transfusion syndrome suggesting that similar circulatory mechanisms may be responsible for defective development, preferably of the upper body parts [41, 42]. The peculiarity of our case 7 lies in the simultaneity of frontal thoracic fusion and lateral cephalic fusion with the two faces displaying two eyes, no nose, two mouths and four ears (parapagous diprosopus). Fusion of two orbits and eyes each, a bilaterally median lower lid notch, arrhinia and microstomia indicated concordant cyclopia (Fig. 5b) and strongly suggested HPE. This combination has not been described before. It raises the question of whether different pathogenic mechanisms are involved that lead to thoracic frontal and facial lateral fusion and to ‘selective ischaemic damage’ of the parasite’s upper body parts.

The *ceramic figurines* of tri-ophthalmic double-faced women (Fig. 1d + e) from the early and middle Formative Mesoamerican period, found in the open cornfields of the Tlatilco village near what is now Mexico City, represent images of an early lethal congenital malformation projected onto a so-called ‘pretty lady’ image, provided with a spiritual exaltation and associated with fertility beliefs. From the accuracy of the portrayals, we can conclude that they are based on real observations [2–5]. The braided hairstyle of the figurine in Fig. 1d (1200–900 B.C.E.) resembles the brain shape in case 4, showing exencephaly with two parallel bars of bulging brain tissue (Fig. 1a). The question arises of whether the seemingly braided coiffure does not in fact refer to an exencephaly, especially since cranial NTDs occur in nearly half of the diprosopus cases. The same applies to the double-faced ceramic figurine in Fig. 1e (500–400 B.C.E.), but with the difference that her head shape is reminiscent of anencephaly with a narrow hair ring between the forehead and the membrana cerebrovasculosa, typically covering the exposed skull base (Fig. 1b). The date of origin of numerous other double-faced Tlatilco figurines found in different geologic strata is given as the early (1800–1200 B.C.E.) and middle (1200–400 B.C.E.) preclassical periods [2–4]. They show various degrees of facial duplication and different hairstyles, referring to different observations. With respect to the higher prevalence of diprosopia in Latin America, it seems worth noting that these early observations concern a pre-Hispanic Mesoamerican population and cultural environment.

In *Schedel's world chronicle*, published in 1493, we found another early illustration of a diprosopus showing a man with four eyes but only one nose and one mouth in the interfacial midline [5] (Fig. 1h). This is certainly not an accurate rendering of 'partial' diprosopus. When two eye pairs are present, one would expect duplication of the forebrain, each developing a set of telencephalic vesicles with a rhinencephalon, that are responsible for the formation of two sets of olfactory placodes in between two lens placodes each, and finally of the localization of two noses in between two eyes each [6] according to the graphics of Foerster [43], Bendersky [2] and Bidondo et al. [1]. Thus, we may assume that the artist had not personally witnessed this event, but rather gained knowledge of it by hearsay, when collecting whimsical phenotypes for his world chronicle.

More difficult is the interpretation of *Paul Klee's* (* 1879) water colour painting of a diprosopus from 1933, which marked the beginning of his 'late work’ in the year of his escape from Germany into his Swiss exile (Fig. 1f). This creative period was overshadowed by a severe sclerodermia that led to a first respiratory crisis in 1935 and to his early death in 1940. The illness gave him the feeling of constriction and disintegration of body parts, known to have influenced his late works. We may thus consider this painting named 'parent's image' (in literal translation 'parent's mirror') as a perceived symbolic facial duplication with hopefully intact central nervous system.

*Conclusion *Brain malformations in patients with diprosopus may not be regarded as an independent mutagenic or teratogenic event but rather as a sequel closely related to the duplication of the notochord and neural plate and as a consequence of the cerebral and associated craniospinal structures. Thus, neural tube closure may be hampered by still-fused incompletely duplicated neural plates, thus explaining the high rate of cranial NTDs. Focal structural instability caused by duplication of bones of the predominantly anterior skull base may give rise to anterior encephaloceles at osseous junctions. HPEs may develop independently from an additional causative HPE gene variant and in Janiceps twins by secondary fusion of primarily cleaved frontal lobes. The aetiology of diprosopus seems complex. The exclusion of a causative gene variant by WES and the occurrence of diprosopia after ICSI in case 3 may be understood as mechanically induced, supporting a non**-**genetic aetiology, but cannot rule out with certainty a monogenic or polygenic cause by an underlying germline or somatic mutation. The association of lateral facial duplication with frontal exoparasitic twinning may indicate the involvement of different pathogenic pathways, including ischaemic damage. With regard to the double-faced Tlatilco figurines, DeMyer's statement *‘the face predicts the brain'* could be modified to state *‘the hairstyle predicts the brain’*.

## Methods

High resolution computed tomography with 3D-reconstructions (3D-CT) was performed on the foetuses in cases 4 and 5 (HRi). In the foetus in case 7, CT could not visualize the skeleton, indicating postmortem removal in accordance with the extended dorsal skin sutures. A corresponding skeletal museum specimen was not found. CT imaging was performed with a third generation Dual Source scanner (Somatom Force, Siemens Healthineers, Erlangen, Germany) in spiral mode with the foetus in the supine position. Imaging parameters were 150 kVp, fixed tube current of 50 mAs, 0.4 mm section thickness, 0.3 mm section interval, ultra-high-resolution kernel (Ur69), iterative reconstruction (Sapphire) strength 5, and transverse orientation of the sections. Multiplanar images of the bones were reconstructed using the postprocessing platform Syngo Via (VB40, Siemens Healthineers, Erlangen, Germany). For 3D visualization, cinematic rendering was applied using environmental lightning for the optimal delineation of all three-dimensional structures.

Molecular analysis by WES in case 3 was performed on DNA derived from foetal muscle tissue (M.S.). Foetal DNA samples were prepared following the workflow of the Twist Library Preparation with Enzymatic Fragmentation and Twist Universal Adapter System (Twist Bioscience; South San Francisco, California, USA). For enrichment of exonic regions the “Twist Human Comprehensive Exome” with standard hybridization was used. The final library was paired-end sequenced on an Illumina NextSeq500 sequencer. The obtained sequencing reads were aligned to GRCh37/hg19 using Burrows Wheeler Aligner (BWA-MEM) and further processed in house according to the GATKs best practice protocol for calling single nucleotide variants, insertions and deletions. Evaluation of the called variants was performed using the program VarSeq from Golden Helix Inc®. Variants were classified according to the American College of Medical Genetics guidelines [44].

## Data Availability

Five double-faced, monocephalic and single-trunk foetuses meeting the criteria of a true diprosopus, and two monocephalic double-faced ventrally fused conjoined twins were included in this study. The foetuses in cases 1, 2, 6 were sent for autopsy to the former central Working Group of Foetal Pathology (WGFP) at the Institute of Pathology in Lübeck in 1977, 1986 and 1980. The foetus in case 3 was sent to the present WGFP at the Institute of Pathology in Marburg in 2013, and three foetuses (cases 4 [1920], 5 [birth date not registered], 7 [1790]) were exhibits from the Fool’s Tower Collection in Vienna. All dicephalic single-trunk foetuses and a pair of synotic monoprosopus Janiceps-like twins were excluded. Autopsies were performed in foetuses 1–3 and 6. Autopsy reports *of the exhibits* were no longer available with the exception of an external description in case 6 in Latin.

## Abbreviations

2(3)DCT: Two(three)dimensional computer tomography
(B)CE: (Before) current era
BWA-MEM: Burrows Wheeler Aligner-MEM
CLP: Cleft lip and palate
CNS: Central nervous system
GATK: Genome analysis toolkit
HPE: Holoprosencephaly
HPO: Human phenotype ontology
ICSI: Intracytoplasmic sperm injection
MAF: Minor allele frequency
MEC: Meningoencephalocele
MRI: Magnetic resonance imaging
NTD: Neural tube defect
OMIM: Online Mendelian Inheritance in Man
SHH: Sonic hedgehog gene
Shh: Sonic hedgehog protein
SIX3: Six homeobox 3 gene
TGIF1: Transforming growth factor-beta-induced factor gene
WES: Whole exome sequencing
ZIC2: ZIC family, member 2 gene

## Availability of data and materials

Data that support the findings of this study are included in this article. Further enquiries can be directed to the corresponding author.
